# Quo Vadis Advanced Prostate Cancer Therapy? Novel Treatment Perspectives and Possible Future Directions

**DOI:** 10.3390/molecules26082228

**Published:** 2021-04-12

**Authors:** Jana Kvízová, Vladimíra Pavlíčková, Eva Kmoníčková, Tomáš Ruml, Silvie Rimpelová

**Affiliations:** 1 Department of Biochemistry and Microbiology, University of Chemistry and Technology, Technická 3, 166 28 Prague, Czech Republic; jakvizka@gmail.com (J.K.); vladimira.pavlickova@vscht.cz (V.P.); tomas.ruml@vscht.cz (T.R.); 2 Bioinova, s.r.o., Vídeňská 1083, 140 20 Praha, Czech Republic; 3 Institute of Experimental Medicine of the Czech Academy of Sciences, Vídeňská 1083, 142 20 Prague, Czech Republic; Eva.Kmonickova@lfp.cuni.cz

**Keywords:** advanced prostate cancer treatment, androgen deprivation therapy, antiandrogen therapy, cancer diagnostics, immunotherapy, multimodal therapy, photodynamic therapy, phototherapy, phototherapy, specific drug targeting

## Abstract

Prostate cancer is a very common disease, which is, unfortunately, often the cause of many male deaths. This is underlined by the fact that the early stages of prostate cancer are often asymptomatic. Therefore, the disease is usually detected and diagnosed at late advanced or even metastasized stages, which are already difficult to treat. Hence, it is important to pursue research and development not only in terms of novel diagnostic methods but also of therapeutic ones, as well as to increase the effectiveness of the treatment by combinational medicinal approach. Therefore, in this review article, we focus on recent approaches and novel potential tools for the treatment of advanced prostate cancer; these include not only androgen deprivation therapy, antiandrogen therapy, photodynamic therapy, photothermal therapy, immunotherapy, multimodal therapy, but also poly(ADP-ribose) polymerase, Akt and cyclin-dependent kinase inhibitors.

## 1. Introduction

Worldwide, the second most frequently diagnosed type of cancer affecting men is prostate cancer. It usually develops very slowly, and the early stages of the disease (curable by prostatectomy, radiotherapy, or active surveillance) are mostly asymptomatic. For this reason, the disease is usually diagnosed only at advanced stages or even late metastatic phases, which are, in most cases, barely treatable, and the average five-year survival prognosis reaches only 30% of the cases. One of the current treatment approaches of advanced prostate cancer focuses on male sex hormones, androgens, which regulate the proliferation of both tumor and normal prostate cells, i.e., starts with androgen deprivation therapy (surgical or chemical castration) followed by antiandrogen therapy. However, the growth of prostate tumor cells is often androgen-independent. Moreover, the effect of hormonal therapy gradually decreases, which frequently results in the development of resistance leading to subsequent progression of the disease. Therefore, searching for novel therapeutic but also diagnostic methods of this disease is inevitable. Two of the relatively novel diagnostic and therapeutic approaches involve photodynamic and photothermal therapy. They are based on the application of a photosensitive agent (photosensitizer, PS), preferably accumulating in rapidly proliferating cells, such as tumor cells. Activation of a photosensitizer by a light source (usually a laser or light-emitting diode) leads to reactive oxygen species formation, which eliminates the tumor in a series of reactions. Advantageously, these approaches can be easily combined with standard treatment methods to significantly improve the therapeutical outcome.

## 2. Prostate Cancer Treatment

### 2.1. Treatment with Antiandrogens

**Figure 1:**
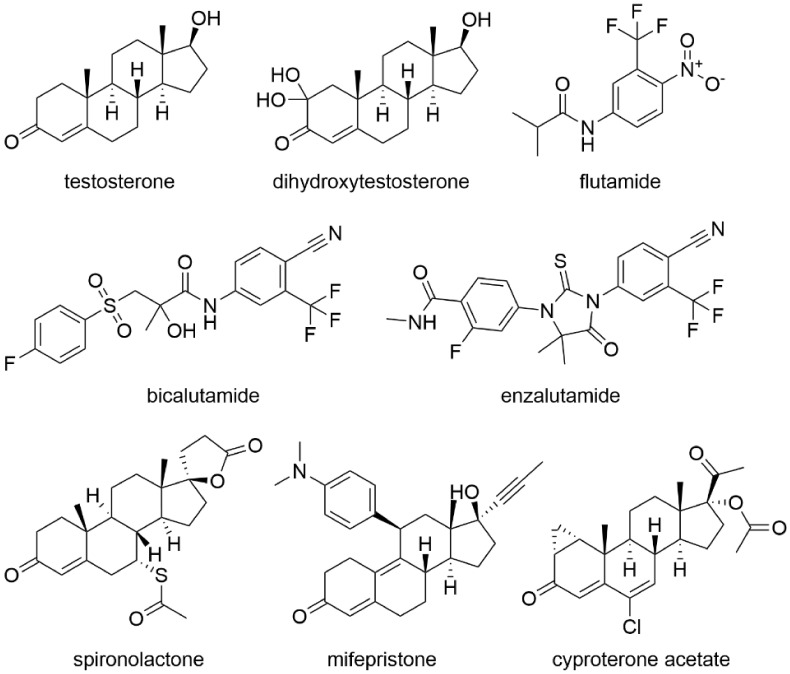
Chemical structures of antiandrogens used in prostate cancer treatment.

The effects of androgens in a cell are mediated by the androgen receptor (AR), the overexpression of which occurs in almost all primary prostate cancers [1,2]. One of the androgens, the male sex hormone testosterone (see the chemical structure in Figure 1), regulates tumor cell growth, and its serum level can be reduced by hormone therapy. The effects of testosterone at both the cellular and molecular levels can be blocked by antiandrogens—drugs structurally similar to androgens. Antiandrogens competitively bind the AR and prevent binding of its natural ligands testosterone and dihydrotestosterone (DHT; chemical structure in Figure 1). Since prostate cancer cells are susceptible to androgen manipulation, reducing the testosterone serum levels to levels present after castration leads to disruption of AR signaling [3,4,5].

**Figure 2:**
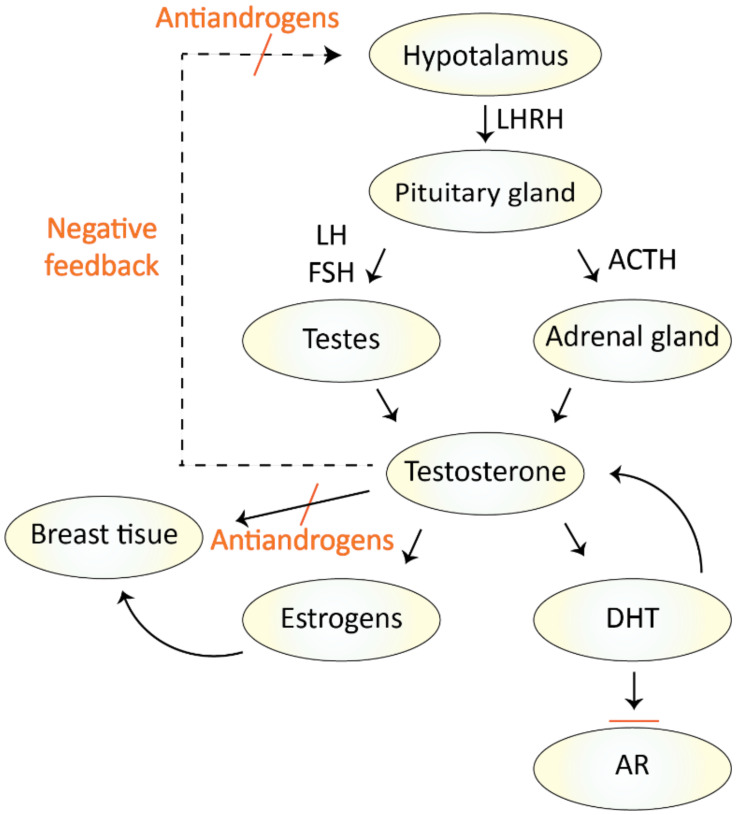
The mechanism of action of nonsteroidal antiandrogens in endocrinologic pathways in men; adapted and adjusted from ref. [6]. LH—luteinizing hormone; LHRH—luteinizing hormone-releasing hormone; FSH—follicle-stimulating hormone; ACTH—adrenocorticotropic hormone; AR—androgen receptor; DHT—dihydrotestosterone.

Antiandrogens can be classified as compounds of steroidal and nonsteroidal character. The nonsteroidal ones (e.g., bicalutamide, flutamide) exhibit only antagonistic activity by which they block the binding of the AR and thereby inhibit tumor growth. However, they do not reduce the testosterone serum levels, unlike steroid molecules [1,6,7]. Regarding the second group of nonsteroidal antiandrogens, they can be further divided into two groups of compounds of the first and second generation (their mechanism of action is outlined in Figure 2). The mechanism of action of the first-generation antiandrogens is based on competitive binding to the AR binding site with testosterone and other androgens. They affect the process of AR translocation into the nucleus and block the downstream signaling and thus cell proliferation, while the second-generation antiandrogens inhibit the androgen biosynthesis [8].

The first-generation antiandrogen used in clinical practice for the longest time has been flutamide (Figure 1) followed by nilutamide. Nevertheless, both of these compounds have begun to be partially replaced by newer nonsteroidal antiandrogens such as bicalutamide (Figure 1), mainly due to improved patient tolerance and overall efficacy [9,10]. The most well-known representative of the second generation of nonsteroidal antiandrogens is enzalutamide (Figure 1) a derivative of diarylthiohydantoin (summarized in Ito and Sadar, 2018 [11]). Its mechanism of action is probably caused by disruption of the AR nuclear translocation via DNA binding, which causes immobilization of regulatory proteins subsequently leading to inhibition of AR target gene transcription and induction of apoptosis. Enzalutamide became in 2012 the first second-generation AR antagonist approved by the US Food and Drug Administration (FDA) for the treatment of patients with castration-resistant prostate cancer (CRPC) [12,13,14,15]. Enzalutamide has a high affinity for the AR, which is eight times higher than that of bicalutamide [14]. Other representatives of the second generation of antiandrogens are apalutamide (summarized in ref. [16]) and darolutamide (summarized in ref. [17]), which have the same mechanism of action as enzalutamide and abiraterone acetate, i.e., they block the androgen biosynthesis by inhibition of cytochrome P450-17A1 (CYP17A1), which, however, leads to significant side effects [18]. While apalutamide is structurally similar to enzalutamide and has a 7–10 times higher affinity for the AR in human cells derived from prostate carcinoma (LNCaP) [19], the structure of darolutamide is unique and varies from other AR antagonists. It is composed of two pharmacologically active diastereoisomers [20].

Unlike nonsteroidal antiandrogens, the steroidal ones have both agonistic and antagonistic androgenic activity which blocks the production of luteinizing hormone and follicle-stimulating hormone, by which they reduce the testosterone serum levels. Another difference between the group of steroidal and nonsteroidal antiandrogens is the fact that they are weak partial agonists of the AR able to activate this receptor and also exhibit progestogenic, glucocorticoid, antimineralocorticoid, and antigonadotropic effects [1,7]. The group of steroidal compounds with antiandrogenic activity also comprises an antagonist of the mineralocorticoid receptor spironolactone (Aldactone^®^; Figure 1), the progesterone receptor antagonists RU-486 (mifepristone, Mifeprex^®^; Figure 1), and an atypical progestin cyproterone acetate (CPA, Androcur^®^; Figure 1) [21].

### 2.2. Treatment with Androgen Deprivation

**Figure 3:**
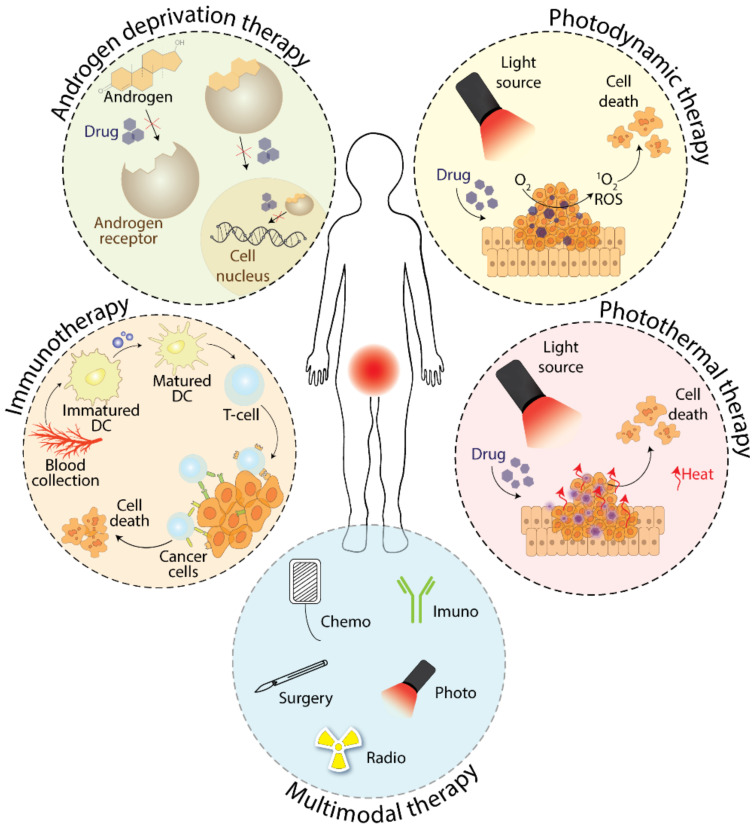
A scheme of various approaches for advanced prostate cancer treatment. DC—dendritic cells.

Nowadays, in addition to antiandrogens, hormonal treatment of prostate cancer comprises also pharmacological castration called androgen deprivation therapy (Figure 3). This method is an alternative to surgical castration and can be achieved by administering agonists or antagonists of luteinizing hormone-releasing hormone (LHRH) [4]. By decreasing the serum level of circulating testosterone, which affects the proliferation and differentiation of prostate cancer cells, it disrupts signaling via the AR [22,23,24]. However, over time, patients develop resistance to androgen-deprivation therapy (ADT), and proliferation of tumor cells occurs even despite the decreased testosterone serum levels, which results in short survival time (usually between 2–4 years) of patients with CRPC [25].

**Figure 4:**
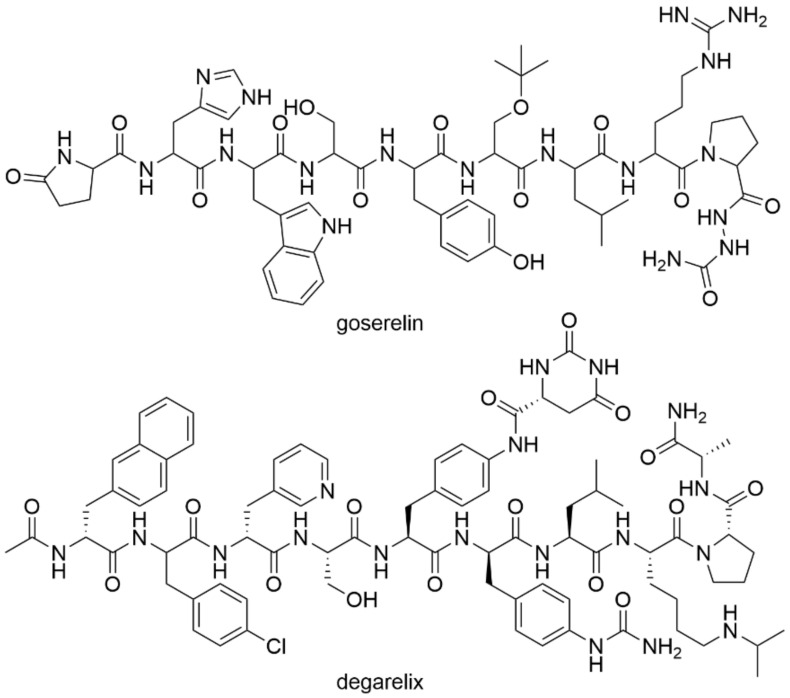
Chemical structures of agonists and antagonists of luteinizing hormone-releasing hormone.

LHRH agonists continuously stimulate the pituitary gland, and thereby suppress the gonadotropin secretion. This results in decreased serum levels of circulating luteinizing hormone (LH), follicle-stimulating hormone (FSH), and testosterone. The LHRH agonists are usually administered either by repeated injections or by depot preparations [26]. Several research studies showed that LHRH analogs also directly inhibit prostate tumors via specific LHRH receptors. An example of an LHRH agonist is the synthetic decapeptide drug goserelin (Figure 4). Goserelin rapidly binds to the LHRH receptor in the pituitary gland, which leads to an initial increase in LH and FSH production, further followed by an increase in serum testosterone levels. As a result, the LHRH agonists may be administered concomitantly with antiandrogens such as flutamide [27]. Goserelin is being used to treat localized prostate carcinoma or for palliative treatment of advanced prostate cancer [28]. An alternative to goserelin is triptorelin. Regarding the survival time of patients with prostate carcinoma, the use of LHRH agonists has been shown to be effective to the same extent as surgical castration [29].

The LHRH antagonists act by rapid inhibition of LHRH without any initial stimulation of the LHRH receptor. The physiological response is immediate and there is a rapid decrease in serum levels of LH, FSH, and testosterone without any initial increase. This is in contrast to the use of LHRH agonists. LHRH antagonists are designed to avoid the stimulation phase of the LHRH receptor [30,31]. An example of an LHRH antagonist is degarelix (Firmagon^®^; Figure 4) approved by the European Medicines Agency (EMA). Degarelix is a selective antagonist of gonadoliberin used to treat adult men with advanced hormone-dependent prostate carcinoma. It competitively and reversibly binds to receptors releasing gonadotropins in the pituitary gland in the brain, thereby rapidly reducing the release of gonadotropins, LH, and FSH, which leads to the reduction of testosterone secretion in the testes [32,33,34,35]. Although LHRH therapy might seem to be effective, it presents one major disadvantage, and this is long-term side toxicity since low testosterone serum levels corresponding to that after castration results in bone demineralization and an increased risk of myocardial infarction and diabetes.

## 3. Phototherapy as a Tool for Prostate Cancer Treatment

A possible alternative to hormonal treatment of prostate cancer could be phototherapy, either photodynamic therapy (PDT) or photothermal therapy (PTT). Both of these approaches utilize a photoactive compound, which itself (without light activation) is nontoxic. Phototherapy offers considerable advantages not only regarding negligible invasiveness, the ability of the photoactive molecule to localize in a tumor without specific/active targeting, which results in low systemic toxicity but also a very low risk of resistance development. Besides, phototherapy occurs only at the site activated by a light source of a given wavelength (summarized in ref. [36]). At the same time, this fact may also be a drawback of this cancer treatment approach due to the difficult activation of a photoactive molecule in solid tumors localized deeper in the tissue [37].

### 3.1. Photodynamic Therapy

PDT (Figure 3) is a relatively new therapeutic and diagnostic method utilizing a PS preferentially accumulating in tumor tissue. In addition, the PS can also be specifically targeted to the diseased tissue, which can significantly increase the efficacy together with diminishing the side effects. After administration, the PS is activated by light and induces a sequence of several reactions that destroy the tumor [38]. During these events, photooxidation of biomolecules may occur by three different mechanisms, according to which PDT can be divided into three subclasses: PDT of the I., II., and III. type. In the first one, upon activation by light of a given wavelength, the PS is excited to the singlet state, from which it subsequently enters the triplet state. Then, by electron transfer from the environment to the PS in the triplet state, there are formed radicals, which subsequently react with the ubiquitous molecules of oxygen (in triplet state) and water. This generates reactive oxygen species (ROS) that react with DNA and lipoproteins, oxidize them as a result of which they destroy the tumor [36]. In the second type of PDT, a PS in the triplet state reacts directly with oxygen molecules to form singlet oxygen. Like ROS, the highly reactive singlet oxygen destroys a tumor in which a PS is localized [39]. The III. type of PDT uses the PS molecule in the triplet state, which does not react with the molecular oxygen but reacts directly with the surrounding biomolecules. The latter mechanism can be very advantageous because larger tumors usually do not have sufficient oxygen supply. In addition to the PDT classification according to its mechanism of action, it can be also divided according to PS targeting, which can be either passive or active.

Passive PS targeting is based on the phenomenon of increased permeability of blood vessels and enhanced PS retention in tumor tissue. Nanoparticles can increase the solubility of often hydrophobic PSs in water, thereby increasing PS accumulation in tumor cells. Alternatively, PS can be modified by ligands increasing cell specificity of PS drugs [40,41]. Such active targeting can be achieved by modification with specific antibodies, nanobodies, affibodies, peptides, aptamers [42], androgens [43], or natural products such as sesquiterpene lactones and their derivatives [44,45,46]. Besides nanoparticles, liposomes are also a viable alternative for PS targeted delivery. Liposomes are formed by one or more concentric phospholipid bilayers containing an aqueous phase within and between the bilayers. The advantage of using liposomes is the possibility to encapsulate both hydrophilic and hydrophobic substances. Liposomes can be also conjugated with a suitable ligand, e.g., antibody, to specifically deliver a PS into target cells [40,47].

In the case of prostate cancer, it is desirable to target the prostate-specific membrane antigen (PSMA) also named N-acetyl-L-aspartyl-L-glutamate peptidase I (NNALADase I) or also glutamate carboxypeptidase II (GCPII). This can be achieved by utilization of a radiolabeled (In^111^) monoclonal antibody, such as capromab pendetide (ProstaScint^®^, CYT-356), which is mainly used for diagnostic imaging by single-photon emission computed tomography (SPECT). Another very promising agent for anticancer therapy which is recently in clinical development is represented by the radiotherapeutic 177-lutetium-PSMA-617 which was designed for prostate cancer cells expressing PSMA. Liu et al. [48] have also focused on the use of PSMA inhibitors conjugated to a PS consisting of pyrophosphoride-α for targeted PDT treatment of prostate cancer. More recent studies are focused on low-molecular-weight PSMA inhibitors linked to a phthalocyanine PS (IRDye700DX). This approach allows prostate tumor resection by using fluorescence image-guided surgery followed by PDT elimination of surgically nonremovable tumor tissue [49]. Another application is a conjugation of a PS with antibodies against the transferrin receptor, which is overexpressed on the surface of many solid tumors [50,51].

### 3.2. Photothermal Therapy

Another approach used to treat prostate tumors is PTT (Figure 3). The principle of PTT lies in heating the diseased tissue to temperatures above the physiological temperature, i.e., up to circa 41–45 °C. At these conditions, the tumor cells are sensitized and, thus, are more susceptible to anti-cancer chemotherapeutics. This approach was described in detail by Zhang et al. [52]. The authors proposed the synthesis and application of pH-responsive nanoparticles coated by a polymer and loaded with doxorubicin. Such chemotherapeutic-loaded nanoparticles exhibited promising results for the elimination of human prostate cancer cells of PC-3, DU145, and LNCaP cell lines. The authors showed that laser irradiation of the nanoparticles significantly increased the overall cytotoxicity and doxorubicin efficacy due to the application of the combined approach of PTT and a chemotherapeutic [52].

Alternatively, to using temperatures in the range of 41–45 °C, PTT can be applied at an even higher temperature of about 47 °C, at which the tumor tissue is directly thermally ablated [36]. Moreover, PTT can be also utilized for controlled drug release at the site of action. The use of nanoparticles that carry a chemotherapeutic drug in their core, seems to be a particularly advantageous approach for cancer treatment. Application of near-infrared irradiation of nanoparticles leads to their degradation and release of encapsulated drugs at the tumor site as a result of which, the side effects on the organism are minimized and cytotoxicity at the tumor site is maximized. This approach was recently successfully applied in vitro in the case of human cells derived from breast, cervical, lung, and prostate carcinoma as well as in mouse cells from prostate carcinoma [53,54,55,56,57,58].

For PTT, the most commonly utilized small organic compounds are derivatives of indocyanine green, cyanines, and phthalocyanines. What is preferred when choosing the proper agent for PTT, is the high absorbance in the near-infrared part of the spectra (approx. 700–1870 nm) [59] since it enables deeper penetration into the tumor tissue. Alternatively, metal nanoparticles composed of gold, palladium, or copper can be also used. From these, for PTT, the most suitable ones are gold nanoparticles (GNPs) since they exhibit optimal photophysical properties [60]. This is mainly due to the phenomenon of localized surface plasmon resonance, which is subsequently relaxed by non-radiative processes in the form of heat that destroys tumor cells. However, the great disadvantage of this method is the frequent aggregation of GNPs, which leads to loss of optical properties. To avoid this undesirable phenomenon, the particles should be further functionalized on their surface, e.g., by using silicon dioxide. This approach was utilized by Kim et al. [61], who prepared silica-coated gold-nanoparticle clusters (AuNC@SiO_2_) for PTT. The AuNC@SiO_2_ exhibited enhanced stability in solution as well as higher efficacy of photothermal transduction than unmodified GNPs tending to agglomerate. Further, the in vitro PTT efficacy of AuNC@SiO_2_ in human prostate carcinoma PC-3 cells was also significantly improved. The PC-3 cells treated with AuNC@SiO_2_ showed a decrease in viability by 80% compared to the untreated control, while non-photoactivated nanoparticles induced only a 20% decrease in cell viability. Moreover, in vivo experiments evidenced a substantial reduction in tumor size after the application of AuNC@SiO_2_ and laser photoactivation. Notably, 15 days after the treatment, the tumors were eliminated [61]. Another valuable tool for targeted PTT of prostate cancer is so-called mesenchymal stem cell-derived gold star nanoparticles produced directly in the cells and secreted in a form of microvesicles based upon a light irradiation stimulus. The main advantage of such nanoparticles is the fact that they exhibit up to 90% efficacy of the photothermal transformation [62,63].

PTT agents that made it to clinical trial evaluation, are gold silica nanoshells, AuroShells^®^ [64,65], prepared for near-infrared laser excitation. In a pilot clinical trial, Rastinehad et al. [66] introduced that these AuroShells^®^ were successfully used in fifteen patients (sixteen lesions) with low and intermediate-grade prostate tumors. After three months, the ablation zones were tumor-negative in 62.5% (10/16) of cases, and after twelve months, 87.5% (14/16) of lesions were negative for a tumor in the ablation zones [66].

## 4. Immunotherapy in the Therapy of Prostate Cancer

Immunotherapy (Figure 3) can also be used to treat prostate cancer, as prostate cancer cells commonly express on their surface several altered antigens, including prostate-specific antigen (PSA), PSMA, or prostatic acid phosphatase (PAP) [67]. The goal of immunotherapy is to overcome the immunosuppressive microenvironment in tumor tissue and to stimulate the patient’s immune self-defensive response against tumor cells [68]. There are many approaches to do this, so only some of them are mentioned. In general, they are either antigen nonspecific (various immune system stimulators) or antigen-specific (targeted to tumor structures). The antigen nonspecific ones include, for example, cytokine administration or nonspecific stimulation of inflammation. While the antigen-specific ones use monoclonal antibodies that specifically target and eliminate cells expressing a certain anti-tumor antigen. These have been the most widely used in clinical practice. Another possibility is to utilize tumor-infiltrating lymphocytes usually obtained directly from a tumor or T-lymphocytes with a chimeric (artificial) T-cell receptor (Chimeric Antigen Receptor T-Cell Therapy, CAR T). However, stimulation (especially of cellular immunity components) and the addition of an effective adjuvant are needed to increase the effect of immunization with the tumor cells alone [69,70]. The need for stimulation and the addition of adjuvants can be eliminated by the administration of dendritic cells (DC), which are used in active cell immunotherapy to produce so-called antitumor vaccines. The production of such vaccines is based on the cultivation of patient peripheral blood monocytes, from which immature DC are prepared in a laboratory. The immature DC are pulse-activated with tumor-associated antigens (peptides, mRNA, apoptotic cells, or cell lysates) that transform them into mature DC, which are then administered back to the patient. A vivid example of such an approach is Sipuleucel-T (Provenge), the only FDA-approved immunotherapy for the treatment of patients with symptomatic or minimally symptomatic CRPC [71,72]. In the Czech Republic, there is undergoing a second phase of clinical trials using an autologous DC activated cell cancer immunotherapy (DCVAC/PCa; Sotio) in patients diagnosed with metastatic CRPC (mCRPC). However, DCVAC is being tested also in early-stage patients. Unlike Sipuleucel-T, DCVAC/PCa utilizes not only a single tumor-associated antigen to pulse DC, but whole cells of the LNCaP prostate cancer cell line [73].

In addition to the aforementioned active immunotherapy, there is also passive immunotherapy based on blocking the inhibitory molecules of the so-called “checkpoint” inhibitors. In particular, these include cytotoxic T-lymphocyte-associated protein 4 (CTLA-4, CD152), the programmed cell death 1 receptor (PD-1 also known as CD279), and ligand 1 and 2 of programmed cell death receptor (PD-L1 and PD-L2 also known as CD274 and CD273, respectively). These molecules appear on activated cytotoxic T lymphocytes and serve to regulate the immune response to prevent autoimmune damage [74,75]. An example of such a molecule is pembrolizumab, which blocks PD-1. This humanized monoclonal antibody was tested in a clinical study for the treatment of prostate adenocarcinoma [76]. Combinations of pembrolizumab with other drugs are also in clinical trials as the effect of monotherapy in prostate cancer treatment seems to be very limited, so far.

## 5. Multimodal Therapy of Prostate Cancer

The treatment of prostate cancer is primarily provided by monotherapy, as mentioned above (e.g., by the administration of antiandrogens or LHRH agonists/antagonists). The problem with monotherapy is that drug resistance occurring with repeated administration reduces its efficacy. The use of a combination of different drugs or treatment approaches (multimodal therapy, Figure 3) may not only help to overcome the resistance, but also lead to a synergistic effect and thus increase the effectiveness of the treatment and improve the overall outcome. Therefore, multimodal therapy is increasingly used today both for the treatment of cancer and for several other diseases [77,78,79].

An example of this strategy is the combination of radiotherapy with long-term androgen deprivation. Which demonstrated an improvement in the overall clinical condition and survival of a patient with advanced prostate cancer compared to radiotherapy alone. The rate of local treatment failure decreased from 34% when using only the radiotherapy to 21 and 15% for short-term and long-term ADT, respectively [80,81].

In multimodal therapy, it is also possible to combine cytostatics with PDT to treat localized and partially metastasized prostate tumors. An example is Tookad^®^ (WST-11), which has progressed to the third phase of clinical trials for PDT targeted to the vascular system. It causes irreversible damage to cell membranes, small arterioles and blocks blood and nutrient supply to tumors. The damage of vascular endothelium triggers a cascade of events leading to tumor necrosis. The principle of this vascular-targeted photodynamic therapy (VTP) is based on the fact that light for PS photoactivation can be delivered by specially developed optical fiber throughout the whole prostate and into metastases in different organs [82,83,84,85]. Unlike chemotherapy or radiotherapy, the mechanism of the cell death induced by PDT is not directly dependent on DNA damage, thereby reducing the possibility of drug resistance, minimizing side effects (photoactivation of only diseased tissue or selective PS targeting), as well as subsequent effects on surrounding “normal” tissue, such as the possible emergence of additional cancerous growth and spreading caused by previous treatment (radiotherapy or chemotherapy) [86].

In addition to PDT, PTT can also be combined with other treatment approaches. This is evidenced by a combination of chemotherapy with PTT, in which polydopamine nanoparticles with poly(allylamine) citraconic anhydride and doxorubicin were successfully applied both in vitro and in vivo. In this study, doxorubicin was released only in the acidic environment of prostate carcinoma [52]. PTT was also successfully combined with immunotherapy, in which a photothermal reagent phthalocyanine IR700 was conjugated to a monoclonal antibody against PSMA [87].

Another possible way of combined therapeutic approaches is chemotherapy and immunotherapy. Indeed, some chemotherapeutics induce so-called immunogenic cell death, which induces an immune response and activates the immune system, thereby enhancing the antitumor immune response [88,89]. Furthermore, chemotherapeutics may interfere with immune mechanisms that suppress the antitumor immunity. This is possible, for example, by reducing the number of regulatory T cells [90]. However, the key to successful treatment is the correct timing of chemotherapy and immunotherapy to create a potentiating effect that can increase treatment success even in patients in more advanced stages of the disease. Such an example would be a study evaluating a combination of a monoclonal fully humanized antibody (PSMA antibody-drug conjugate) raised against the PSMA tumor antigen together with a conjugated cytostatic inhibiting microtubule polymerization (monomethyl-auristatin E; for details on mitotic poisons, see review in ref. [91]) that binds to PSMA positive tumor cells and results in a cytotoxic effect. The results of the study showed a decrease in the PSA levels and a decrease in the number of circulating tumor cells [92].

However, multimodal therapy does not have to lie only in a combination of two treatment approaches but, as it is shown in the study of Tekin et al. [93], it can involve even a higher number of different methods as documented by ^89^Zr-Pt@TiO_2_-SPHINX nanoconjugates. ^89^Zr-Pt@TiO_2_-SPHINX particles serve not only for in vivo imaging of cancer cells using positron emission tomography and optical imaging but combine also a photosensitizer and a radiosensitizer. Moreover, it was found out that LNCaP and PC-3 cancer cell treatment with these nanoconjugates induces enhanced expression of an antiangiogenic splicing isoform of the vascular endothelial growth factor (VGFA), VGFA_165_b, which can further lead to suppression of both angiogenesis and metastasis [93].

## 6. Poly(ADP-Ribose) Polymerase Inhibitors

Since circa 15–25% of men suffering from prostate cancer have similar defects in DNA repair, they could benefit from treatment with poly(ADP-ribose) polymerase inhibitors (PARPi). PARPi are a group of antineoplastic drugs that were developed for the treatment of patients diagnosed with mutations in the breast cancer type 1 susceptibility protein 1 and 2 (BRCA1/2) genes, i.e., for breast and ovarian cancer patients. The mechanism of PARPi action is the blockade of the enzyme poly(ADP-ribose) polymerase (PARP), which is commonly involved in the repair of DNA breaks, including homologous recombination repair, non-homologous end-joining, and base-excision repair [94,95,96]. It was reported that cells bearing the BRCA1/2 tumor suppressor gene deletion/mutation are markedly more sensitive to PARP inhibition than cells not expressing this gene. When BRCA and PARP genes are co-deleted, these cells experience an increase in further mutations due to the formation of double-stranded DNA breaks, leading to cell cycle arrest and cell death. This process is also referred to as “synthetic lethality” [97]. Synthetic lethality happens when PARPi are co-administered with another drug/agent or are in a combination with the occurrence of a genetic alteration in cancer cells. This leads to DNA damage and results in cell death.

Recently, it was shown that the same PARPi could be a valuable tool also for the treatment of advanced and metastasized prostate cancer (15–25% of mCRPC have homologous recombination deficiency). One of the PARPi, olaparib, was approved by the FDA in 2020 for the treatment of patients with the germinal and or somatic mutation BRCA1/2 of mCRPC. In addition, olaparib is also approved for patients who have experienced one of the other twelve homologous recombination-associated changes in the genome in ATM (ataxia-telangiesctasia mutated), BARD1 (BRCA1-associated RING domain protein 1), BRIP1 (Fanconi anemia group J protein), CDK12 (cyclin-dependent kinase 12), CHEK1 and CHEK2 (checkpoint kinase 1 and 2), FANCL (E3 ubiquitin-protein ligase FANCL), PALB2 (partner and localizer of BRCA2), RAD51B (DNA repair protein RAD51 homologue 2), RAD51C (DNA repair protein RAD51 homolog 3), RAD51D (DNA repair protein RAD51 homolog 4), or RAD54L (DNA repair and recombination protein RAD54-like). PARPi rucaparib has been approved by the FDA for the treatment of patients with mCRPC and BRCA1/2 mutations who have progressed after previous treatment with second-generation anti-AR drugs or taxane-based chemotherapy. It is currently in the third phase of clinical trials (NCT02975934) [98], at which the most commonly reported adverse effects following PARPi administration were anemia, fatigue, nausea, and dyspnoea.

In connection to prostate cancer, several other clinical studies investigating the effects of PARPi such as niraparib (phase III, NCT03748641) [99], talazoparib [100] have recently been terminated or are still ongoing (phase III, NCT03395197) [101], veliparib (phase II, NCT01576172, terminated 2020) [102], pamiparib (phase II, NCT03712930, terminated 2020) [103,104] or rucaparib (phase I, NCT03572478) [105]. In these clinical studies, PARPi are usually co-administered with other, commonly used, drugs such as abiraterone acetate, prednisone, enzalutamide, and nivolumab [106,107,108]. These studies have been performed in both patients bearing and not bearing mutations in the DNA repair mechanisms. An overview of clinical trials was reported by Nizialek and Antonarakis [109], and Powers et al. [110]. Therapies using PARPi in combination with immunotherapy, radiotherapy, and drugs targeting AR are also being studied, see ref. [111]. A summary of ongoing and completed clinical trials on PARPi for prostate cancer treatment is given in Table 1.

## 7. Akt Inhibitors

Protein kinase B (Akt) plays a key role in cellular signal transduction, in which it is responsible for the biological response upon activation of transmembrane receptors. It is part of the PI3K/Akt/mTOR signaling pathway (phosphatidylinositol-3-kinase/protein kinase B/mammalian target of rapamycin), which is an important target of antitumor therapy. In connection with prostate cancer, changes in the genome of the PI3K/Akt/mTOR pathway were detected in up to 40% of early diagnosed cases and 70% of patients with advanced cancer [113,114]. This signaling pathway begins with the binding of a ligand to PI3K, which is converted to phosphatidylinositol-3,4,5-trisphosphate (PIP_3_). This step is commonly regulated by the phosphatase and tensin homologue (PTEN), which catalyzes the transition of phosphatidyl triphosphate to phosphatidyl-4,5-bisphosphate, thereby inactivating it. This is followed by Akt binding to activated PIP_3_, which is thereby phosphorylated. The activated Akt is involved in several cellular processes such as mTOR activation and the associated proteosynthesis, cell proliferation, regulation of angiogenesis and cell cycle, but also glucose metabolism. In the presence of mutations such as, for example, PTEN deletion, Akt hyperactivation occurs which has been linked to increased resistance to chemotherapy and radiotherapy, thus resulting in a worse prognosis and shorter patient survival rate [113,115,116]. Inhibition of any step of the PI3K/Akt/mTOR pathway thus appeared to be a vital way to anticancer drug development.

However, the results of initial studies showed that when used as monotherapy, inhibitors of the individual steps of this cascade did not provide the desired results and exhibited high systemic toxicity, therefore, many studies were terminated after the first or second phase of clinical trials. It turned out that the idea of a linear view on PI3K/Akt/mTOR cascade does not correspond to reality, which is much more complex, since the whole signaling pathway intersects with other signaling pathways at many points. These intersections can compensate for the steps blocked by the PI3K/Akt/mTOR pathway inhibitors and the therapy is thus ineffective. This was recently summarized in detail in ref. [114]. The proposed solution is to combine inhibitors of the PI3K/Akt/mTOR pathway with another drug, such as abiraterone acetate. An ATP-competitive inhibitor of Akt, ipatasertib co-administered with abiraterone acetate successfully passed the second phase of clinical trials; an increase in radiographic progression-free survival was observed in patients with mCRPC, especially in patients diagnosed with PTEN loss [117]. In this combination, ipatasertib was shifted to the third phase of clinical trials (NCT03072238) for the treatment of mCRPC [118].

In addition to the aforementioned study, ipatasertib has entered several other clinical studies in combination therapy, which are currently in the first phase of evaluation. In combination with atezolizumab (a monoclonal antibody against the programmed cell death-ligand 1), ipatasertib is being evaluated in the IceCAP study (NCT03673787, phase I/II) for the treatment of advanced solid tumors, glioblastoma multiforme, and prostate cancer [119]. Another study applying combination therapy for the treatment of mCRPC verified the effect of ipatasertib co-administered with atezolizumab and docetaxel (NCT04404140) [120]. For the treatment of metastatic hormone-sensitive prostate cancer, another Akt kinase inhibitor capivasertib co-administered with abiraterone acetate is in the third phase of clinical trials (NCT04493853) [121]. The first phase of clinical trials is the combination therapy of capivasertib with abiraterone acetate or enzalutamide for mCRPC treatment (NCT04087174) [122]. Further, a recently completed ProCAID study (Phase II, NCT02121639) [123] on capivasertib co-administered with docetaxel and prednisolone did not prolong progression-free survival of patients with mCRPC, as it was reported for triple-negative breast cancer, even though that in both studies, the target was the PI3K/AKT/PTEN pathway [124,125]. From these results, it has been shown that also other genes, such as the estrogen receptor or receptor tyrosine-protein kinase erbB-2 (HER2/neu), play a significant role in the disease, and that it is crucial to properly select the patient group with a known tumor genome to achieve successful treatment outcomes. A summary of ongoing and completed clinical trials on Akt inhibitors for prostate cancer treatment is given in Table 2.

## 8. Cyclin-Dependent Kinase Inhibitors

Other potential targets to treat prostate cancer include cyclin-dependent kinase (CDK) inhibitors. These enzymes are involved in the regulation of the cell cycle and cell growth. In the case of carcinogenesis, this pathway is often disrupted, leading to uncontrolled cell proliferation. The most common targets are CDK4 and CDK6, which are involved in the transition from G1 to the S phase of the cell cycle. Several CDK inhibitors (e.g., palbociclib, ribociclib, abemaciclib) have been currently approved by the FDA for the treatment of metastatic breast cancer. Palbociclib is also currently being tested in several clinical trials for the treatment of prostate cancer, and it is being evaluated in the second phase of clinical trials as monotherapy for mCRPC (NCT02905318) [126]. In addition, palbociclib combined with androgen deprivation therapy is in the second phase of clinical trials for the treatment of retinoblastoma protein-positive metastatic prostate cancer (NCT02059213) [127]. However, more detailed results of this study have not yet been published [128,129]. For patients with hormone-resistant and metastatic prostate cancer, ribociclib co-administered with enzalutamide (NCT02555189) is in the second phase of clinical trials [130]. Similarly, ribociclib is also in the second phase of testing co-administered with docetaxel and prednisone for the treatment of mCRPC (NCT02494921) [131]. Further, patients with mCRPC previously treated hormonally or with taxane chemotherapy are in the second phase of clinical trials with applied abemaciclib monotherapy (NCT04408924) [132]. In several studies, it is then androgen deprivation therapy combined with radiotherapy in the second phase of clinical trials (RAD 1805, NCT04298983) [133]. For the treatment of mCRPC, abiterone acetate is combined with prednisone (NCT03706365) [134] or with immunotherapy in a form of abemaciclib (NCT04751929) [135]. A summary of ongoing and completed clinical trials on CDK inhibitors for prostate cancer treatment is given in Table 3.

## 9. Conclusions

Prostate cancer is one of the most common cancers in men and is asymptomatic in the early stages of the disease. By the time the disease is detected, the vast majority of prostate tumors are still sensitive to hormonal treatment, but over time, the natural progression of the disease leads to the development of hormone-independent prostate cancer. From the above, it is clear that the standard hormone deprivation therapy in prostate cancer treatment has its limits. Therefore, it is important to develop and implement novel therapeutic approaches, such as PDT, PTT, or immunotherapy as well as combining these new ways of therapy into a multimodal therapy. This might significantly improve the treatment of advanced prostate cancer and, thus, increase the overall effectiveness of the therapy, reduce systemic toxicity and resistance to the treatment.

## Figures and Tables

**Table 1:** A summary of ongoing or completed clinical trials using poly(ADP-ribose) polymerase (PARPi) in prostate cancer treatment, registered to April 2021 in ref. [112].

PARPi	Combination	Indication	Status	Phase	Clinical Trial Identifier
Olaparib	Monotherapy	mCRPC, a mutation in one of 15 genes involved in the HRR	n.r.	III	NTC02987543
	Monotherapy	Advanced CRPC	n.r.	II	NCT01682772
	Monotherapy	mCRPC, after docetaxel treatment	n.r.	II	NCT03434158
	Monotherapy	Non-mBRPC	Recruiting	II	NCT03047135
	Prior to radical prostatectomy	localized PC	Terminated	II	NCT03570476
	Prior to radical prostatectomy	Locally advanced PC with DNA repair defects	Recruiting	II	NCT03432897
	Ceralasertib	mCRPC, with DNA repair-deficient	Recruiting	II	NCT03787680
	Bromodomain and extraterminal inhibitor (AZD5153)	Malignant solid tumors	n.r.	I	NCT03205176
	AA	mCRPC, prior chemotherapy containing docetaxel	n.r.	II	NCT01972217
	AA, P	mCRPC, with DNA repair-deficient	Recruiting	II	NCT03012321
	PLX2853, AA, P	mCRPC	Recruiting	Ib/IIa	NCT04556617
	MEDI4736, cediranib	Advanced solid tumors	Recruiting	I/II	NCT02484404
	AA	mCRPC, no prior chemotherapy or new hormonal agents	n.r.	III	NCT03732820
	Testosterone	mCRPC	n.r.	II	NCT03516812
	Degarelix, prior to radical prostatectomy	Intermediate/high risk PC	Completed	I	NCT02324998
	Cediranib	mCRPC	n.r.	II	NCT02893917
	Nanoparticle camptothecin	mCRPC, relapsed/refractory small cell lung cancer	Recruiting	I/II	NCT02769962
	Carboplatin	mCRPC, tumors containing BRCA1, BRCA2 or PALB2	Recruiting	II	NCT04038502
	Carboplatin, cabazitaxel, P	Aggressive variant PC	n.r.	II	NCT03263650
	Pembrolizumab	mCRPC, failed to respond to either AA or enzalutamide (but not both), and chemotherapy	Recruiting	III	NCT03834519
	Radiation, ADT, AA, P	CSOmPC	n.y.r.	II	NCT04748042
	Pembrolizumab	mCRPC	Recruiting	Ib/II	NCT02861573
	Durvalumab	CSBR non-mPC, harboring mutations in DNA damage repair	Recruiting	II	NCT03810105
	^177^Lu-PSMA	mCRPC	Recruiting	I	NCT03874884
	Ra-223 dichloride	mCRPC	Recruiting	I/II	NCT03317392
Rucaparib	Monotherapy	mCRPC, HRR gene deficiency	Recruiting	III	NCT02975934
	Monotherapy	mHSPC	Recruiting	II	NCT03413995
	Monotherapy	Non-mHSPC	Recruiting		NCT03533946
	Monotherapy	mCRPC, ovarian, epithelial, peritoneal, fallopian tube cancer	n.y.r.	III	NCT04676334
	Monotherapy	Solid tumors associated with deleterious mutations in HRR genes	Recruiting	II	NCT04171700
	Monotherapy	mCRPC	n.r.		NCT02952534
	Nivolumab	mCRPC	n.r.	II	NCT03338790
	Nivolumab	PC, endometrial cancer	Terminated	Ib/IIa	NCT03572478
	Ipatasertib	Advanced BC, OC, PC	n.r.	I/II	NCT03840200
	Enzalutamide, AA	mCRPC	n.r.	Ib	NCT04179396
	Enzalutamide	mCRPC, resistant to testosterone-deprivation therapy	Suspended	III	NCT04455750
	Docetaxel, carboplatin	mCRPC, HRR gene deficiency	Recruiting	II	NCT03442556
	Copanlisib	mCRPC	Recruiting	Ib/II	NCT04253262
Niraparib	AA, P	mCRPC, HRR gene alteration	Recruiting	III	NCT03748641
	AA, P	mCSPC, deleterious germline or somatic HRR gene-mutated	Recruiting	III	NCT04497844
	AA, P	mCRPC, with and without HRR gene alterations	Recruiting	I	NCT04577833
	Cetrelimab, AA, P	mCRPC	Recruiting	I/II	NCT03431350
	Monotherapy	Platinum sensitive CRPC	Recruiting	II	NCT04288687
	Apalutamide, AA, P	mCRPC	Completed	I	NCT02924766
	Monotherapy	mCRPC and DNA repair anomalies	n.r.	II	NCT02854436
	Monotherapy	Advanced solid tumor or hematologic malignancies	Completed	I	NCT00749502
	Prostatectomy	Non-metastatic high-risk PC	Recruiting	II	NCT04030559
	Cabazitaxel, carboplatin, cetrelimab	Aggressive variant mPC	Recruiting	II	NCT04592237
	Leuprolide, AA, P, radiotherapy	High risk and node-positive PC	Recruiting	I/II	NCT04194554
	ATR inhibitor (BAY 1895344)	Advanced solid tumors, OC	Recruiting	Ib	NCT04267939
	Radium 223	mPC, hormone refractory PC, stage IV PC	n.r.	Ib	NCT03076203
	Radiotherapy, antiandrogen therapy	high-risk PC	Recruiting	II	NCT04037254
Talazoparib	Monotherapy	mCRPC, previously received taxane-based chemotherapy and progressed on at least 1 novel hormonal agent	n.r.	II	NCT03148795
	Monotherapy	Advanced cancer, a mutation in DNA damage response genes	Recruiting	II	NCT04550494
	Enzalutamide	mCRPC	Recruiting	III	NCT03395197
	Enzalutamide	mHSPC	Recruiting	II	NCT04332744
	Enzalutamide	mCSPC	n.y.r.	III	NCT04821622
	Glutaminase inhibitor telaglenastat	mCRPC	n.y.r.	II	NCT04824937
	Temozolomide	PC	Recruiting	Ib/II	NCT04019327
	belinostat	mCRPC, mBC, OC	n.y.r.	I	NCT04703920
	ADT	mCSPC	n.y.r.	II	NCT04734730
	Avelumab, bempegaldesleukin	Advanced squamous cell carcinoma of the head and neck, mCRPC	Terminated	II	NCT04052204
	Avelumab	Advanced or metastatic solid tumors	n.r.	Ib/II	NCT03330405
	Monotherapy	Advanced or recurrent solid tumors	Completed	I	NCT01286987
	Monotherapy	Metastatic solid tumors	Withdrawn	I	NCT02567396
Veliparib	AA, P	mCRPC	Completed	II	NCT01576172
	Temozolomide	mPC	Completed	I	NCT01085422
	Monotherapy	Malignant solid tumors	Completed	I	NCT00892736
Pamiparib	Monotherapy	mCRPC	Terminated	II	NCT03712930
	Temozolomide	Locally advanced or metastatic solid tumors	Recruiting	I/II	NCT03150810

AA—abiraterone acetate; ADT—androgen-deprivation therapy; CSOPC—castration sensitive oligometastatic prostate cancer; BC—breast cancer; CSBR non-mPC—castration sensitive biochemically-recurrent non-metastatic prostate cancer; HRR—homologous recombination repair; ^117^Lu-PSMA—^177^lutetium-prostate-specific membrane antigen; mBRPC—metastatic biochemically-recurrent prostate cancer; MEDI4736—anti-programmed death ligand-1 antibody; mHSPC—metastatic hormon sensitive prostate cancer; n.r.—not recruiting; n.y.r.—not yet recruiting; OC—ovarian cancer; P—prednisone; PLX2853—non-benzodiazepine bromodomain and extraterminal domain inhibitor; PC—prostate cancer; mPC—metastatic prostate cancer; ^223^Ra—radium-223.

**Table 2:** List of clinical trials on protein kinase B inhibitors (Akt) in connection with prostate cancer treatment, posted in last ten years (2011–2021), data are from ref. [112].

Akt Inhibitor	Combination	Indication	Status	Phase	Clinical Trial Identifier
Ipatasertib	AA	mCRP	n.r.	III	NCT03072238
	AA	mCRP, previous treatment with docetaxel	n.r.	I/II	NCT01485861
	Androgen deprivation therapy, darolutamide	Localized, high-risk PC	n.y.r.	I/II	NCT04737109
	Atezolizumab	Advanced solid tumors, glioblastoma multiforme, PC	Recruiting	I/II	NCT03673787
	Atezolizumab, docetaxel	mCRPC previously treated with second-generation AR targeted therapy	Recruiting	Ib	NCT04404140
	Rucaparib	Advanced BC, OC, PC	n.r.	I/II	NCT03840200
	Monotherapy	PC	Recruiting	II	NCT03385655
	Monotherapy	Solid tumors, lymphomas, multiple myeloma			
Capivasertib	AA, P	mHSPC	Recruiting	III	NCT04493853
	AA, enzalutamide	mCRPC	n.r.	I	NCT04087174
	Monotherapy	Solid tumors, lymphomas, multiple myeloma	Recruiting	II	NCT02465060
	Docetaxel, P	mCRPC	n.r.	I/II	NCT02121639
	Monotherapy	mCRPC	Completed	I	NCT01692262
	Enzalutamid	mCRPC	Unknown	II	NCT02525068
	Enzalutamide, fulVestrant	Advanced solid tumors (PC, BC)	n.r.	I	NCT03310541
	Itraconazole	Healthy volunteers (intended indication: metastatic triple negative/HR^+^ BC, mHSPC)	n.r.	I	NCT04712396

AA—abiraterone acetate; Akt—protein kinase B; mHSPC—metastatic hormone-sensitive prostate cancer; BC—breast cancer; P—prednisone; PC—prostate cancer; mCRPC—metastatic castration-resistant prostate cancer; n.r.—not recruiting; n.y.r.—not yet recruiting.

**Table 3:** List of clinical trials on cyclin-dependent kinase (CDK) inhibitors in connection with prostate cancer treatment, posted in last ten years (2011–2021), data are from ref. [112].

CDK Inhibitor	Combination	Indication	Status	Phase	Clinical Trial Indetifier
Palbociclib	Monotherapy	mCRPC	Recruiting	II	NCT02905318
	bicalutemide, zoladex, lupron depot	Retinoblastoma protein-positive mPC	Completed	II	NCT02059213
	KAT6 inhibitor (PF-07248144)	Advanced or metastatic solid tumors (BC, PC, lung cancer)	Recruiting	I	NCT04606446
	Monotherapy	Advanced refractory solid tumors, lymphomas, multiple myeloma	Recruiting	II	NCT02465060
	Combinations of drug	Metastatic solid tumor or hematological malignancy	Recruiting	Ib	NCT03878524
Ribociclib	Enzalutamide	HRPC, mPC	Recruiting	Ib/II	NCT02555189
	Docetaxel, P	mCRPC	n.r.	II	NCT02494921
Abemaciclib	Monotherapy	mCRPC previously treated hormonally or with taxane chemotherapy	Recruiting	II	NCT04408924
	Androgen deprivation therapy, radiation therapy	Locally advanced PC	Recruiting	II	NCT04298983
	Atezolizumab	mCRPC	Withdrawn	II	NCT04272645
	AA, P	mCRPC	n.r.	II	NCT03706365
	Atezolizumab	mCRPC	n.y.r.	II	NCT04751929
	Combinations of drug	Metastatic solid tumor or hematological malignancy	Recruiting	Ib	NCT03878524

AA—abiraterone acetate; BC—breast cancer; HRPC—hormone-resistant prostate cancer; P—prednisone; PC—prostate cancer; mPC—metastatic prostate cancer; mCRPC—metastatic castration-resistant prostate cancer; n.r.—not recruiting; n.y.r.—not yet recruiting.

## Data Availability

Not applicable.

## Abbreviations

ADT	Androgen-deprivation therapy
Akt	Protein kinase B
AR	The androgen receptor
AuNC SiO_2_	Silica-coated gold-nanoparticle clusters
CAR T	Chimeric antigen receptor T-cell therapy
CDK	Cyclin-dependent kinase
CPA	Cyproterone acetate
CRPC	Castration-resistant prostate cancer
CTLA-4	Cytotoxic T-lymphocyte antigen 4 (CD152)
CYP17A1	Cytochrome P450-17A1
DC	Dendritic cells
DCVAC/PCa	Autologous active cellular immunotherapy based on activated dendritic cells
DHT	Dihydroxytestosterone
DU145	Human cells from prostate carcinoma
EMA	European medicines agency
FDA	U.S. Food and Drug Administration
FSH	Follicle-stimulating hormone
GCPII	Glutamate carboxypeptidase II
GNPs	Gold nanoparticles
HER2/neu	Receptor tyrosine-protein kinase erbB-2
LH	Luteinizing hormone
LHRH	Luteinizing hormone-releasing hormone
LNCaP	Human cells from prostate carcinoma
mCRPC	Metastatic CRPC
NNALADase I	N-acetyl-L-aspartyl-L-glutamate peptidase I
PAP	Prostatic acid phosphatase
PARP	Poly(ADP-ribose) polymerase
PARPi	Poly(ADP-ribose) polymerase inhibitors
PC-3	Human cells from prostate carcinoma
PD-1	Programmed cell death 1 receptor (CD279)
PD-L1	Ligand 1 of programmed cell death receptor
PD-L2	Ligand 2 of programmed cell death receptor
PDT	Photodynamic therapy
PIP_3_	Phosphatidylinositol-3,4,5-trisphosphate
PS	Photosensitizer
PSA	Prostate-specific antigen
PSMA	Prostate-specific membrane antigen
PTEN	Phosphatase and tensin homologue
PTT	Photothermal therapy
ROS	Reactive oxygen species
SPECT	Single-photon emission computed tomography
VGFA	Vascular endothelial growth factor
VTP	Vascular-targeted photodynamic therapy
